# Wearing Protection or Wearing Assumptions? A Critical Reappraisal of the Wearable Cardioverter-Defibrillator

**DOI:** 10.3390/jcm15218358

**Published:** 2026-10-27

**Authors:** Francesco De Sensi, Pasquale Baratta, Ugo Limbruno

**Affiliations:** Azienda Unità Sanitaria Locale Toscana Sud Est, Misericordia Hospital, Via Senese 161, 58100 Grosseto, Italy

**Keywords:** LifeVest, wearable cardioverter-defibrillator (WCD), implantable cardioverter-defibrillator (ICD), VEST trial, WEARIT, SCD-PROTECT

## Abstract

The wearable cardioverter-defibrillator (WCD) has become an accepted temporary strategy for patients considered at transient risk of sudden cardiac death and its use has rapidly expanded across a broad range of clinical settings. However, more than twenty years after its introduction, the evidence supporting its routine use remains surprisingly limited. This review revisits the background rationale, randomized evidence and observational literature. Rather than questioning the technical ability of the WCD to terminate ventricular arrhythmias, we critically examine whether available evidence demonstrates a meaningful improvement in prognosis. We discuss the lessons learned from early post-myocardial infarction Implantable Cardioverter Defibrillator (ICD) trials, the limitations of using appropriate shocks as surrogate endpoints, the interpretation of the VEST trial, the strengths and weaknesses of registry-based evidence, patient adherence and quality-of-life issues, the economic implications of WCD therapy, and the need for a more selective, evidence-based use of the WCD that identifies patients most likely to derive meaningful clinical benefit while promoting responsible stewardship of healthcare resources.

## 1. Introduction

The wearable cardioverter-defibrillator (WCD) was introduced to bridge periods during which patients are considered at increased risk of sudden cardiac death but either are not yet candidates for permanent Implantable Cardioverter Defibrillator (ICD) implantation or cannot undergo ICD implantation despite an established indication, for example after device extraction because of infection or while awaiting heart transplantation. Although this concept is biologically appealing, biological plausibility should not be confused with clinical proof. Current recommendations remain cautious, assigning a Class IIb indication and not recommending routine use of the WCD in the early post-MI phase, but only in selected patients deemed to be at high risk for Sudden Cardiac Death (SCD) [1]. Conversely, over the years, the use of the wearable cardioverter-defibrillator has progressively expanded across a broad range of clinical scenarios, encompassing nearly all patients with newly diagnosed severe left ventricular systolic dysfunction, irrespective of the underlying etiology [2]. This apparent discrepancy between widespread clinical adoption and limited high-level evidence deserves careful examination.

It is important to distinguish primary from secondary prevention. In patients with an established indication for ICD implantation in whom device implantation must be temporarily deferred—for example after extraction of an infected ICD system or while awaiting heart transplantation—the use of the WCD as a bridging strategy is widely accepted because no equivalent alternative for sudden death protection exists. Although some observational registries include such patients, they represent only a minority of the evidence discussed in this Viewpoint. Our critical appraisal is directed principally at the use of the WCD in primary prevention, where indications have expanded considerably despite a substantially more controversial evidence base. The purpose of this review is to critically reappraise the scientific evidence supporting its use and to identify the unresolved questions that should guide future research.

## 2. Literature Search and Evidence Selection

This article was conceived as a critical narrative review. Relevant literature was identified through searches of PubMed/MEDLINE using combinations of the terms “wearable cardioverter-defibrillator”, “WCD”, “LifeVest”, “sudden cardiac death”, “ventricular arrhythmias”, “myocardial infarction”, “heart failure”, “ICD”, “ICD programming”, “VEST”, “registry”, “adherence”, and “cost-effectiveness”. Reference lists of key studies, reviews, consensus documents, and current guidelines were also examined to identify additional relevant publications. Priority was given to randomized trials, major prospective and retrospective WCD registries, contemporary guideline and consensus documents, and studies directly addressing the interpretation of ventricular arrhythmias, adherence, clinical outcomes, and cost-effectiveness. Study selection was guided by relevance to the specific clinical and methodological questions addressed in this critical appraisal rather than by predefined systematic-review eligibility criteria.

## 3. The Early Post-Infarction Paradox: High Arrhythmic Risk, Limited Defibrillator Benefit

The early phase after acute myocardial infarction represents an apparently compelling target for temporary protection against sudden cardiac death. The incidence of sudden death is highest during the first weeks after infarction, particularly among patients with severe left ventricular systolic dysfunction, heart failure, or other markers of electrical instability [3]. This temporal clustering of events provides the intuitive rationale for offering defibrillator protection precisely during the interval in which permanent implantable cardioverter-defibrillator implantation is conventionally deferred. However, the assumption that a higher early incidence of arrhythmic death necessarily identifies a period in which defibrillator therapy improves survival is not supported by randomized evidence. Evidence supporting this concept of competing mortality risk derives primarily from earlier randomized trials of ICD therapy for primary prevention in the early post-myocardial infarction setting.

This apparent paradox was first exposed by the Defibrillator in Acute Myocardial Infarction Trial (DINAMIT). The trial enrolled 674 patients 6 to 40 days after myocardial infarction who had a left ventricular ejection fraction of 35% or less together with impaired cardiac autonomic function. Patients were randomized to prophylactic ICD implantation or conventional medical therapy. During a mean follow-up of 30 months, ICD therapy substantially reduced arrhythmic mortality: 12 arrhythmic deaths occurred in the ICD group compared with 29 in the control group, corresponding to a hazard ratio of 0.42. Nevertheless, this reduction was entirely offset by a higher incidence of non-arrhythmic death, and all-cause mortality was virtually identical between treatment groups [4,5]. The Immediate Risk Stratification Improves Survival (IRIS) trial subsequently reproduced the same finding in a different high-risk population. IRIS randomized 898 patients early after myocardial infarction on the basis of reduced left ventricular ejection fraction combined with an elevated resting heart rate or nonsustained ventricular tachycardia. During a mean follow-up of 37 months, overall mortality was not reduced by ICD implantation: 116 patients died in the ICD group and 117 in the control group, yielding a hazard ratio of 1.04. Sudden cardiac death was less frequent with the ICD, but nonsudden cardiac death was correspondingly more common [6].

However, the distinction between arrhythmic and non-arrhythmic death should be interpreted with caution, as cause-of-death adjudication after myocardial infarction may be uncertain, particularly for unwitnessed events. Thus, while DINAMIT and IRIS robustly demonstrated the absence of an overall survival benefit from early ICD implantation, the reciprocal changes in arrhythmic and non-arrhythmic mortality should not be interpreted as definitive evidence of a shift in the mechanism of death, but rather as reflecting the coexistence of substantial competing mortality risks.

It should be noted that competing causes of death were handled differently across the two pivotal trials. In the original DINAMIT analysis, cause-specific mortality was assessed using Kaplan–Meier methods [4], although a subsequent dedicated competing-risk analysis confirmed a reduction in arrhythmic death with ICD therapy without a corresponding reduction in non-arrhythmic death [5]. In contrast, IRIS formally treated causes of death as competing events using proportional subdistribution hazard models [6]. Thus, although competing-risk methodology was not uniform across the two studies, both consistently showed the same clinically relevant pattern: a reduction in arrhythmic mortality without a corresponding reduction in all-cause mortality.

Rather, both trials demonstrated a reduction in sudden arrhythmic death without a corresponding improvement in overall survival, highlighting the impact of competing risks during the early post-infarction period. Patients at highest risk of ventricular tachyarrhythmias frequently share the same substrate that predisposes them to cardiogenic shock, progressive heart failure, recurrent ischaemia, or other fatal cardiovascular events. Consequently, successful defibrillation may alter the mode of death without changing the overall disease trajectory. This concept is particularly relevant soon after myocardial infarction, when left ventricular dysfunction often reflects a dynamic and potentially reversible process, with substantial recovery following revascularization and guideline-directed medical therapy (Figure 1).

These findings from ICD trials provide a conceptual framework for interpreting WCD therapy in the same vulnerable clinical setting. Although the wearable approach avoids the procedural implications of early permanent ICD implantation and provides temporary, reversible protection, it does not modify the competing non-arrhythmic risks that characterize the early post-infarction period. Therefore, successful termination of a ventricular arrhythmia by the WCD should not necessarily be assumed to translate into improved overall survival.

## 4. Rethinking Ventricular Arrhythmias: Lessons from Contemporary ICD Programming

Appropriate device therapy is frequently interpreted as a surrogate for aborted sudden cardiac death. This assumption is clinically intuitive but methodologically imprecise. Ventricular fibrillation is generally incompatible with sustained circulation if untreated, whereas ventricular tachycardia (VT) comprises a heterogeneous spectrum of rhythms with different rates, substrates, haemodynamic consequences, and spontaneous trajectories. Successful termination of a ventricular tachyarrhythmia therefore establishes technical efficacy, but does not demonstrate that death would otherwise have occurred. Evidence from contemporary ICD programming studies provides an important perspective for interpreting ventricular arrhythmias detected by wearable cardioverter-defibrillators. Over the past two decades, a series of landmark trials has progressively challenged the traditional assumption that every rapid ventricular arrhythmia requires immediate therapy.

PREPARE first demonstrated that prolonging arrhythmia detection and adopting less aggressive programming almost halved the proportion of patients receiving shocks during the first year (9% vs. 17%; *p* < 0.01), without increasing arrhythmic syncope or untreated sustained ventricular arrhythmias [7]. These findings were subsequently confirmed by MADIT-RIT, in which both high-rate detection and delayed therapy markedly reduced inappropriate interventions, while high-rate programming was associated with a 55% reduction in all-cause mortality (HR 0.45; *p* = 0.01), establishing conservative programming as the contemporary standard [8]. A subsequent MADIT-RIT analysis provided important mechanistic insight by demonstrating that prolonging the detection interval allowed many ventricular arrhythmias to terminate spontaneously, while those that persisted continued to receive appropriate therapy without an increase in syncope or mortality [9]. ADVANCE III further translated this concept into clinical practice, demonstrating that prolonged detection significantly reduced ICD therapies (IRR 0.63; *p* < 0.001) without compromising patient safety [10]. Finally, PainFREE Rx II showed that anti-tachycardia pacing successfully terminated approximately three-quarters of fast ventricular tachycardias (188–250 beats/min), avoiding immediate shocks without compromising safety [11]. Collectively, these studies fundamentally changed ICD programming and suggest that many ventricular tachyarrhythmias are neither immediately fatal nor invariably require instantaneous defibrillation. Consequently, the occurrence of a ventricular arrhythmia should not automatically be equated with a life saved (see Table 1 for details).

Direct support for this concept also comes from a WCD population. In WEARIT-II-EUROPE, 10 of 781 patients (1.3%) received appropriate shocks for 13 VT/VF episodes, all successfully terminated with a single shock. In parallel, 22 patients deliberately withheld 47 impending therapies by pressing the response buttons; electrogram review showed that 24 episodes were sustained ventricular tachycardias occurring in 12 patients, while the remainder represented atrial tachyarrhythmias. These observations indicate that a proportion of WCD-detected sustained VTs remained haemodynamically tolerated long enough for patients to consciously abort therapy. As an observational registry, WEARIT-II-EUROPE cannot establish the counterfactual outcome of untreated episodes, but its findings are consistent with evidence from contemporary ICD programming studies [12]. Unlike an ICD, the WCD has no antitachycardia pacing capability and delivers a shock whenever a ventricular tachyarrhythmia persists through the programmed response interval without patient intervention. Consequently, appropriate shocks are frequently used as a surrogate for lives saved, although this assumption cannot be directly verified. Some treated arrhythmias would likely have progressed to ventricular fibrillation, whereas others may have terminated spontaneously, remained haemodynamically tolerated, or allowed sufficient time for emergency medical intervention. Landmark primary-prevention trials such as MADIT II and SCD-HeFT demonstrated that ICD therapy improves survival, but they also highlight the distinction between successful arrhythmia termination and proven mortality reduction [13,14]. Accordingly, appropriate WCD shocks should be interpreted as evidence of successful arrhythmia detection and treatment rather than as a one-to-one estimate of prevented deaths.

## 5. Successful Defibrillation Does Not Necessarily Improve Prognosis

The VEST trial provides a valuable framework to distinguish technical success from clinical benefit. Among 1524 patients randomized to the wearable cardioverter-defibrillator (WCD), 20 patients (1.3%) received at least one appropriate shock and all documented ventricular tachyarrhythmias were initially converted. Nevertheless, only 14 of these 20 patients were alive at 90 days. Thus, 6 (30%) died during the short trial follow-up despite successful arrhythmia termination [15]. This observation does not imply that the shocks were futile; rather, it demonstrates that successful defibrillation and subsequent survival are not interchangeable outcomes.

The individual event data are particularly informative. Of the 48 deaths occurring in the WCD group, only 12 patients were wearing the device at the time of death or at the event leading to death. Among the 25 deaths adjudicated as arrhythmic, nine patients were wearing the WCD. Four of these nine received appropriate shocks for ventricular tachycardia or ventricular fibrillation, with initial conversion to sinus rhythm, but subsequently died after recurrent VT/VF or progression to an agonal rhythm. The remaining 5 patients had no ventricular tachyarrhythmia recorded by the WCD at the time of the fatal event (Figure 2).

These findings show that even when the device is present, activated, and technically effective, the terminal pathway may extend beyond the shockable rhythm itself. This distinction is clinically relevant because sudden cardiac death is not a single electrophysiological event but the final result of a dynamic sequence. In a patient with extensive myocardial injury early after infarction, electrical conversion may therefore restore an organized rhythm without restoring durable mechanical function or altering the underlying trajectory toward death. The 4 VEST patients who initially converted after appropriate therapy but nonetheless died provide direct trial-level evidence of this limitation. Furthermore, five of nine patients classified as having an arrhythmic death while wearing the WCD had no ventricular tachyarrhythmia documented by the device. The subsequent VEST compliance analysis explicitly acknowledged that cause-of-death adjudication is difficult when deaths are unwitnessed or poorly documented and noted that WCD electrograms were not used by the blinded adjudication committee. Accordingly, some events labeled as arrhythmic may have reflected bradyasystolic, pulseless electrical activity, or other non-shockable mechanisms [16].

## 6. The Evidence Gap: Beyond the Randomized Trial

Across WEARIT/BIROAD [17], WEARIT-II [18], WEARIT-France [19], WEARIT-II-Europe [12] and, most recently, SCD-PROTECT [20], the device reliably identified sustained ventricular tachyarrhythmias, delivered effective shocks, produced relatively few inappropriate therapies, and could be used for several weeks or months while ventricular function and the need for a permanent implantable cardioverter-defibrillator (ICD) were reassessed. These are important achievements. They demonstrate technical efficacy and real-world feasibility, and they support the use of the WCD as a temporary monitoring and treatment platform in selected patients.

Importantly, the consistency of these findings across multiple independent cohorts should not be underestimated. Collectively, contemporary registries provide robust real-world evidence that the WCD can reliably detect and successfully terminate potentially life-threatening ventricular tachyarrhythmias, while maintaining a low incidence of inappropriate shocks. These findings represent an important and clinically meaningful strength of WCD therapy, particularly when temporary arrhythmic protection is required.

The absolute event rates, however, are more informative than the commonly reported annualized incidences. In VEST, 20 of 1524 patients assigned to the WCD arm received at least one appropriate shock during approximately three months of follow-up, corresponding to 1.3%, while 9 patients (0.6%) received an inappropriate shock [15]. In WEARIT-II, 22 of 2000 patients (1.1%) received 30 appropriate therapies during a median wear duration of 90 days [18]. WEARIT-France reported appropriate shocks in 18 of 1157 patients (1.6%) during a median wear period of 62 days and inappropriate shocks in 8 patients (0.7%) [19]. In WEARIT-II-Europe, 10 of 781 patients (1.3%) received 13 appropriate shocks [12]. A 2023 updated systematic review, which included one randomized trial and 11 prospective observational studies, found that 1.0% to 4.8% of patients received at least one appropriate shock and that inappropriate shocks occurred in 0% to 2% of patients [21]. Thus, despite differences in populations, indications and analytic methods, the largest contemporary cohorts generally converge on a simple clinical estimate: approximately 1 to 2 patients per 100 prescribed a WCD receive an appropriate shock during a typical two- to three-month period (Figure 3).

Taken together, these registries provide valuable evidence of the technical performance of the WCD, including arrhythmia detection, shock delivery, first-shock success, inappropriate therapy, daily wear time, and recovery of left ventricular ejection fraction (LVEF). However, evidence of technical performance should be clearly distinguished from evidence of long-term clinical benefit. In the absence of an appropriate control group, observational registries are inherently less suited to determining whether successful detection and termination of ventricular arrhythmias translate into a causal reduction in arrhythmic or all-cause mortality. This limitation also arises from the way patients enter observational studies. WCD recipients are not randomly selected from all patients with reduced LVEF; they are chosen because clinicians perceive a sufficiently high or uncertain arrhythmic risk and because the patient is considered able and willing to use the device. The measured incidence of ventricular arrhythmias therefore reflects not only disease biology but also local prescribing practice, referral patterns and clinical judgement. This confounding by indication is amplified when registries combine heterogeneous populations—recent myocardial infarction, newly diagnosed non-ischaemic cardiomyopathy, myocarditis, ICD explantation, transplant candidates and inherited arrhythmic disorders—whose baseline arrhythmic risks, potential for ventricular recovery, and competing modes of death differ substantially. The 2023 systematic review accordingly judged the certainty of observational evidence to be very low and noted that mixed indications limit indication-specific conclusions [21]. Therefore, event rates and WCD treatment rates derived from heterogeneous registry populations should be interpreted within the specific clinical indication and should not be directly extrapolated across different patient groups. Survivor selection and competing risks further complicate interpretation. WEARIT-France is illustrative: 24 of 1157 patients (2.1%) died during WCD use, whereas only 18 patients received an appropriate shock. Such data emphasize that arrhythmic events represent only one component of overall short-term risk [19].

SCD-PROTECT represents the most comprehensive contemporary observational dataset available [20]. The study consecutively included 19,598 German patients prescribed a WCD for newly diagnosed non-ischaemic cardiomyopathy or myocardial infarction/coronary artery disease. Median wear duration was 62 days in the non-ischaemic group and 61 days in the ischaemic group, with median daily use exceeding 23 h. The incidence rate of a first appropriate treatment was reported as 6.10 and 8.64 events per 100 patient-years, respectively, and the overall incidence density of all appropriate treatments as 8.53 and 14.98 per 100 patient-years. Annualized rates are statistically appropriate for person-time analyses, but they can appear more dramatic than the absolute clinical experience because exposure lasted only about two months. The cumulative incidence of patients receiving at least one appropriate treatment was 1.3%—essentially identical to VEST and WEARIT-II-Europe and close to WEARIT-II and WEARIT-France [12,15,18,19] (Figure 4).

Inappropriate shocks occurred in 105 of 19,598 patients (0.5%). Interestingly, total mortality during WCD use was only 154 patients (0.8%), substantially lower than the 3.1% all-cause mortality reported in the VEST trial. Furthermore, LVEF improved to >35% in 52.8% of patients with available follow-up, while only 36.2% ultimately underwent ICD implantation. Taken together, these findings suggest that many patients followed a relatively favorable clinical trajectory, reinforcing the concept that ventricular dysfunction frequently improves during guideline-directed medical therapy and that a large proportion of patients may ultimately avoid permanent ICD implantation.

## 7. Compliance: Why Definitions Matter

Adherence introduces an additional layer of selection. Patients who wear the WCD consistently are likely to differ systematically from those who do not. They may be more adherent to beta-blockers, renin–angiotensin system inhibitors and follow-up visits, more engaged in rehabilitation, and more responsive to medical advice [22]. In WEARIT-France, patients reported that WCD prescription encouraged better self-care and adherence to lifestyle recommendations, illustrating that device use may cluster with other favourable behaviours [19]. This healthy-adherer effect cannot be separated reliably from any true effect of the device in uncontrolled analyses [23]. The VEST per-protocol and as-treated analyses explicitly acknowledged this problem, together with effect–cause bias arising when sicker or hospitalized patients removed the device during periods of higher mortality risk. These analyses are clinically suggestive, but they do not restore the protection against confounding conferred by randomization [16].

The WCD is unique because its effectiveness depends almost entirely on patient behaviour. Continuous wear is required to achieve protection, yet prolonged use may impair sleep, body image, physical activity and cause cutaneous rashes and irritation [15]. Anxiety, alarm fatigue and treatment burden deserve greater attention when evaluating the overall clinical value of the device.

A superficial comparison of reported daily wear times may suggest substantially poorer WCD adherence in VEST than in observational registries such as WEARIT-France. However, this apparent difference is largely driven by fundamentally different definitions of adherence. In VEST, wear time was evaluated over the prespecified study period after randomization, including all patients assigned to the WCD arm and counting days without device use—including days after premature discontinuation—as zero-hour days. Consequently, overall median wear time was 18 h/day. Importantly, the VEST wear-time curves demonstrate that this value combines two distinct dimensions of adherence: the proportion of patients continuing to use the device progressively declined throughout the 90-day follow-up, whereas, among patients who were still wearing the WCD on a given day, daily wear time remained consistently high at approximately 23 h. Accordingly, the overall reduction in median wear time primarily reflects treatment discontinuation rather than poor daily adherence among active users. This interpretation is further supported by the bimodal distribution of wear time, characterized by a predominance of observations clustered around either 0 or 24 h per day, with relatively few intermediate values [15].

By contrast, in WEARIT-France, adherence was calculated only during the period in which the device was actually worn, from the first day of use until permanent discontinuation. Consequently, days occurring after WCD discontinuation were not included in the denominator, and the reported median wear time of 23.4 h/day reflects the intensity of device use among patients remaining on therapy rather than persistence throughout a predefined follow-up period. Therefore, the 23.4 h/day reported in WEARIT-France is directly comparable to the approximately 23 h/day observed among active users in VEST, but not to the overall median of 18 h/day reported in the intention-to-treat analysis. These findings indicate that the apparent discrepancy between the two studies is largely methodological and arises from different definitions of adherence rather than from fundamentally different patient behaviours (Figure 5) [19].

This concept is further supported by the subsequent per-protocol analysis of the VEST trial. In this analysis, patients randomized to the WCD arm were censored on the last day they wore the device, thereby restricting the analysis to the period of actual treatment exposure. Using this approach, a significant reduction in both all-cause mortality (HR 0.25, 95% CI 0.13–0.48; *p* < 0.001) and arrhythmic mortality (HR 0.38, 95% CI 0.17–0.86; *p* = 0.02) was observed. Nevertheless, actual WCD exposure after randomization is not itself a randomized variable. Censoring patients when they discontinue the device therefore compromises the balance achieved by the original randomization and may introduce substantial selection bias. In particular, patients who continue wearing the WCD may also be intrinsically more adherent to pharmacological therapy, medical follow-up, and lifestyle recommendations, reflecting the well-recognized propensity-to-adhere bias. Consequently, although the per-protocol findings are clinically relevant, they should be interpreted with caution and cannot establish a causal mortality benefit of WCD therapy. Rather, it highlights that the duration of actual treatment exposure—and, consequently, the methodology used to define adherence—can substantially influence the interpretation of study outcomes. This observation reinforces the need for caution when directly comparing adherence data across randomized trials and observational registries using different methodological frameworks [16].

## 8. Cost-Effectiveness: Evidence, Assumptions, and the Importance of Patient Selection

Cost-effectiveness analyses supporting WCD use are largely based on decision-analytic models incorporating data on global mortality reduction and assumptions regarding early ICD implantation, device efficacy, healthcare costs, and quality-adjusted life-years (QALYs), rather than reduction in sudden cardiac death [24]. Consequently, their conclusions are highly dependent on the validity of these assumptions. As a simplified clinical and practical illustration of resource utilization, we considered the approximate monthly rental cost of the WCD in our healthcare setting together with the rate of appropriate therapies reported in the literature. Assuming a monthly rental cost of approximately €3000 per patient and, for illustrative purposes, an appropriate therapy rate of 1%, treating 100 patients for one month would correspond to an expenditure of approximately €300,000 per appropriate WCD therapy observed. This calculation is intended solely as a pragmatic illustration and not as a formal or generalizable measure of cost-effectiveness, since device costs, treatment duration, and appropriate therapy rates may vary substantially across healthcare systems and patient populations. Importantly, it refers to the cost per appropriate therapy delivered rather than the cost per life saved. Appropriate shocks represent a surrogate endpoint and cannot be assumed to correspond directly to lives saved, since ventricular arrhythmias may be self-terminating, successfully treated by emergency medical services, or followed by death from progressive heart failure or other competing causes. Accordingly, the frequency of appropriate therapies should not be interpreted as a direct measure of survival benefit or as a standalone measure of cost-effectiveness.

This concept is further reinforced by the sensitivity analyses of Sanders et al., which demonstrated that the economic performance of the WCD is determined primarily by the baseline risk of sudden cardiac arrest rather than by the device itself. When the estimated 3-month risk of sudden cardiac arrest was 4%, the incremental cost-effectiveness ratio (ICER) was approximately US$42,000 per QALY, a value generally considered acceptable within conventional willingness-to-pay thresholds. However, when the estimated risk declined to 0.5%, the ICER increased to more than US$200,000 per QALY, exceeding commonly accepted thresholds for cost-effectiveness [25]. These findings suggest that cost-effectiveness is not an intrinsic property of the WCD, but rather a consequence of appropriate risk enrichment of the treated population. In other words, the economic value of the device depends less on its acquisition cost than on the ability to identify patients with a sufficiently high short-term arrhythmic risk to derive a measurable clinical benefit. Accordingly, broader use of the WCD in populations with a low incidence of treatable ventricular arrhythmias may reduce its economic efficiency, although the magnitude of this effect is likely to vary according to baseline arrhythmic risk, treatment duration, device costs, and healthcare system. These considerations suggest that appropriate patient selection is likely to represent an important determinant of WCD cost-effectiveness. Conversely, WCD use may represent a cost-favorable strategy in carefully selected patients at increased arrhythmic risk, by providing temporary protection while allowing sufficient time for reassessment of ICD eligibility. In this context, Dell’Era et al. reported that 50% of patients managed with a WCD recovered left ventricular ejection fraction and avoided permanent ICD implantation, with an estimated 19.9% reduction in short-term costs compared with an early ICD implantation strategy [26].

## 9. Future Directions

Future investigations should move beyond left ventricular ejection fraction as the predominant criterion for temporary defibrillator therapy. Rather than identifying patients at high overall mortality risk, the key challenge is to identify those with a transiently increased risk of shockable ventricular arrhythmias whose outcome may actually be modified by prompt defibrillation. Multiparametric risk stratification integrating cardiac magnetic resonance imaging and scar characterization, completeness of revascularization, non-sustained ventricular arrhythmic burden, autonomic and electrocardiographic markers, circulating biomarkers, and potentially artificial intelligence-based prediction models may provide a more biologically meaningful assessment of arrhythmic vulnerability. Importantly, such approaches should account for the dynamic nature of arrhythmic risk, which may substantially evolve during myocardial recovery and optimization of medical therapy. Future prospective studies should therefore focus not only on predicting ventricular arrhythmias, but on distinguishing defibrillation-responsive arrhythmic risk from competing non-arrhythmic mortality, thereby identifying the subgroup in whom temporary WCD protection is most likely to translate into a true survival benefit.

## 10. Conclusions

The central question is no longer whether the wearable cardioverter-defibrillator can terminate ventricular arrhythmias. Rather, it is whether current evidence convincingly identifies the patients in whom this intervention changes prognosis. The limitations of the available evidence should not, however, be interpreted as evidence against WCD use. Contemporary registries consistently demonstrate that the device can reliably detect and terminate potentially life-threatening ventricular arrhythmias, with a low incidence of inappropriate shocks, supporting its role as a temporary protective strategy in appropriately selected patients. Twenty years after its introduction, the field has accumulated an impressive amount of observational experience but only limited randomized evidence. A critical reappraisal of the available data is therefore timely and should stimulate a new generation of studies focused on individualized risk stratification rather than broad empirical prescription.

## Figures and Tables

**Figure 1:**
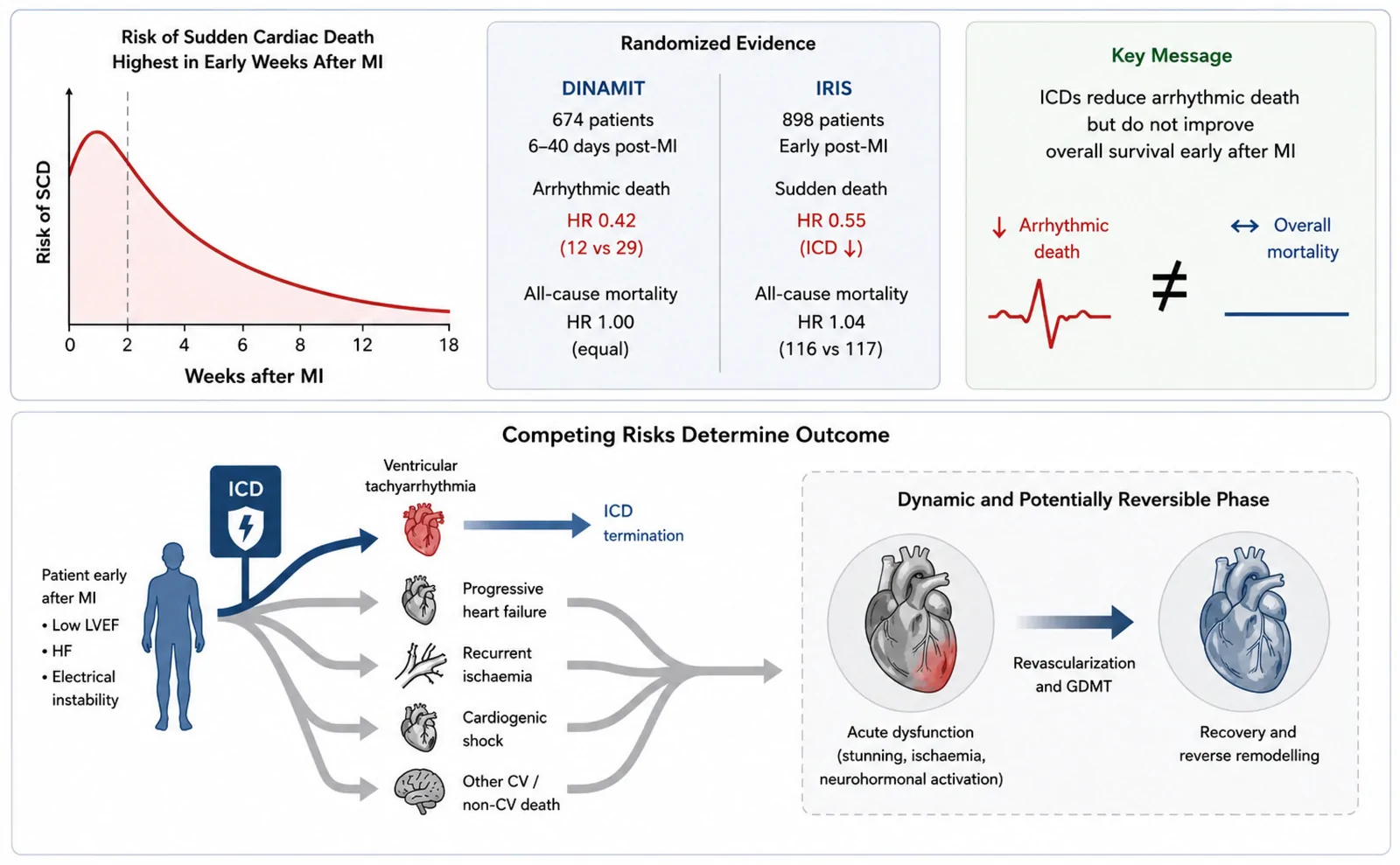
The Early Post-MI Paradox: ICD Reduces Sudden Death, Not Overall Mortality. The early post-MI period is characterized by a high risk of sudden cardiac death but also by competing non-arrhythmic causes of mortality. As demonstrated by the DINAMIT and IRIS trials, ICD therapy reduces arrhythmic death without improving overall survival, while optimization of revascularization and guideline-directed medical therapy may promote recovery during this dynamic and potentially reversible phase. Abbreviations: CV, cardiovascular; GDMT, guideline-directed medical therapy; HF, heart failure; HR, hazard ratio; ICD, implantable cardioverter-defibrillator; LVEF, left ventricular ejection fraction; MI, myocardial infarction; SCD, sudden cardiac death.

**Figure 2:**
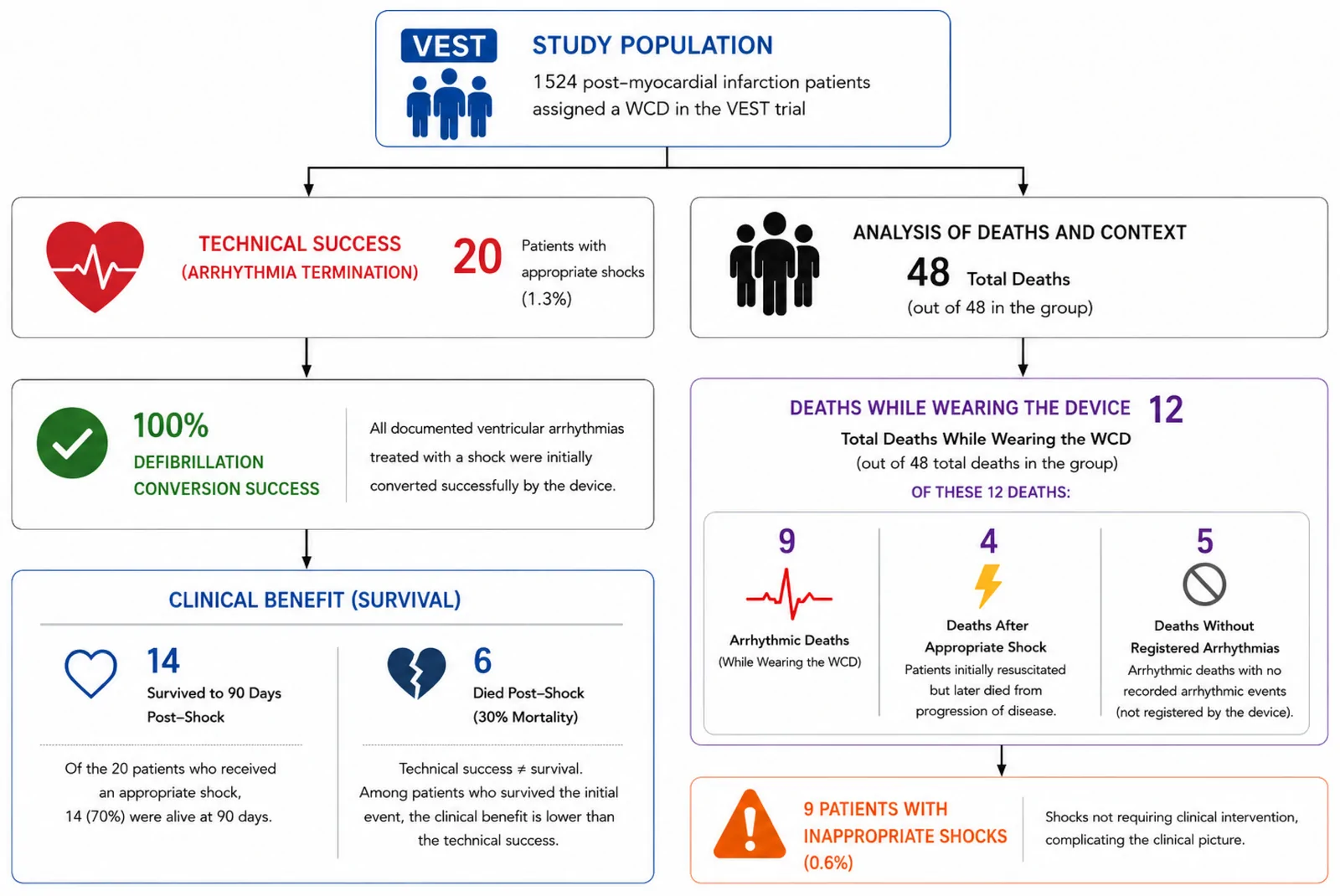
Technical success does not necessarily translate into clinical benefit: lessons from the VEST trial. The VEST trial illustrates the distinction between technical success and clinical benefit. Despite complete first-shock defibrillation success for documented ventricular arrhythmias, survival was influenced by post-resuscitation deaths, deaths without documented ventricular arrhythmias while wearing the device, and inappropriate shocks. These findings emphasize that successful arrhythmia termination should not be interpreted as a direct surrogate for improved survival. Abbreviations: MI, myocardial infarction; VEST, Vest Prevention of Early Sudden Death Trial; WCD, wearable cardioverter-defibrillator.

**Figure 3:**
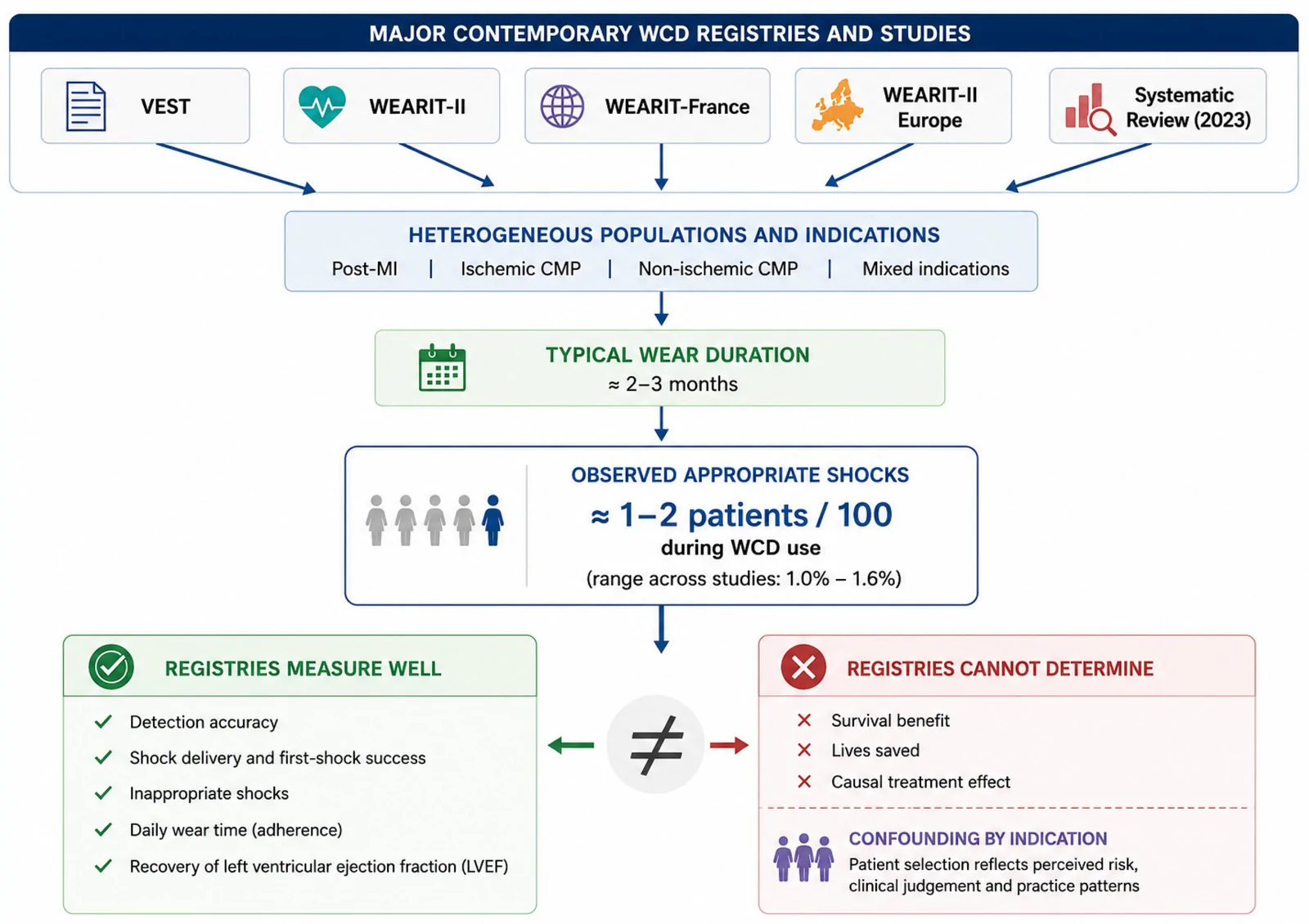
Contemporary WCD registries consistently demonstrate technical device performance but cannot establish clinical benefit. Despite differences in study design, patient populations and indications, contemporary WCD registries consistently report low absolute rates of appropriate shocks during typical wear periods. While these studies reliably assess device performance and safety, they cannot determine the causal effect of WCD therapy on survival because of confounding by indication and the absence of randomized controls. Abbreviations: CMP, cardiomyopathy; LVEF, left ventricular ejection fraction; MI, myocardial infarction; VEST, Vest Prevention of Early Sudden Death Trial; WCD, wearable cardioverter-defibrillator.

**Figure 4:**
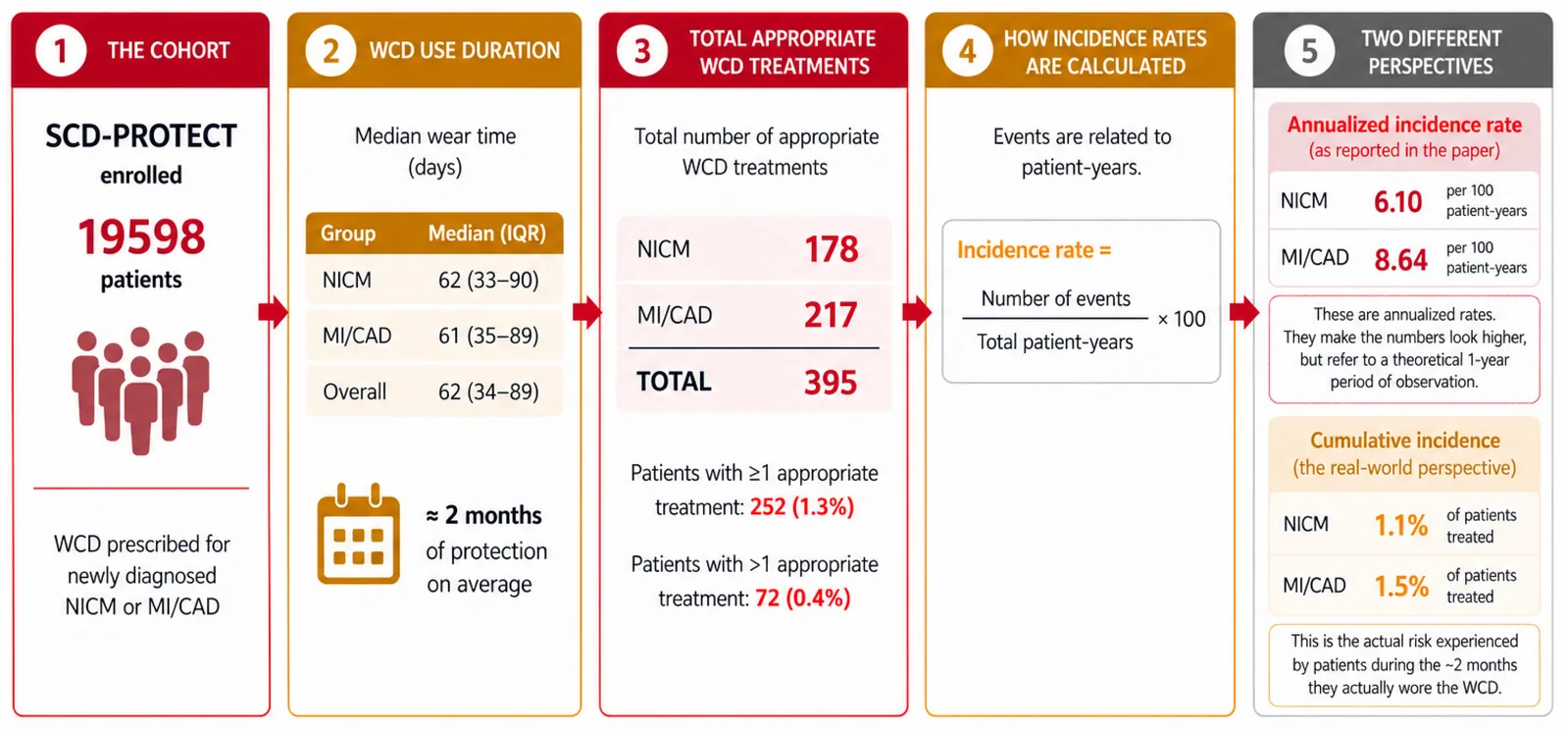
SCD-PROTECT: from annualized incidence rates to the real clinical picture. This figure highlights the distinction between annualized event rates and cumulative incidence, providing a more intuitive estimate of the real clinical burden. Abbreviations: MI/CAD, myocardial infarction/coronary artery disease; NICM, non-ischaemic cardiomyopathy; SCD, sudden cardiac death; WCD, wearable cardioverter-defibrillator.

**Figure 5:**
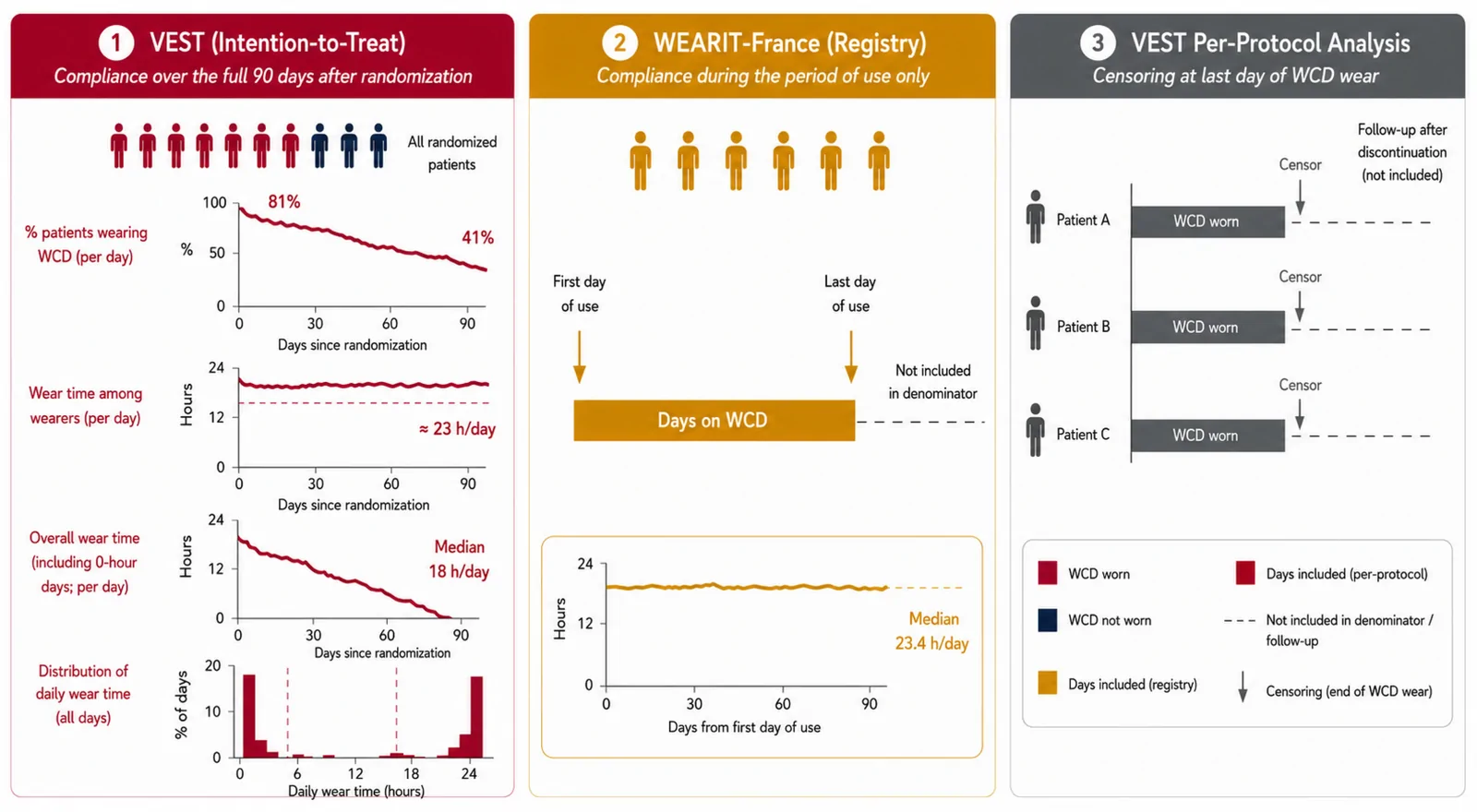
Different definitions of WCD compliance lead to different conclusions. The apparent differences in WCD adherence largely reflect how compliance is defined and analyzed rather than true differences in patient behavior. The VEST intention-to-treat analysis, the WEARIT-France registry, and the VEST per-protocol analysis illustrate three distinct methodological approaches to measuring device use. Abbreviations: WCD, wearable cardioverter-defibrillator.

**Table 1:** Landmark ICD programming trials and their implications for the interpretation of WCD therapies.

Study	Population	Programming Strategy	Main Efficacy Findings	Safety Findings	Clinical Implication for WCD
**PREPARE [7]**	700 primary-prevention ICD recipients	Long detection (30/40), SVT discriminators, ATP before shock	Morbidity index reduced (0.26 vs. 0.69 events/patient-year); shock rate 9% vs. 17% (*p* < 0.01)	No increase in arrhythmic syncope or untreated sustained VT/VF	Many ventricular arrhythmias can be safely observed before therapy
**MADIT-RIT [8]**	1500 primary-prevention ICD recipients	Conventional vs. high-rate (≥200 bpm) vs delayed therapy	Inappropriate therapies reduced (HR 0.21 and 0.24; both *p* < 0.001); all-cause mortality reduced with high-rate programming (HR 0.45; *p* = 0.01)	No increase in syncope	Less aggressive programming improves both efficacy and safety
**MADIT-RIT mechanistic analysis [9]**	Ventricular arrhythmias ≥ 200 bpm	Analysis according to programming strategy	Similar final shock rates despite progressively less ATP delivery	Demonstrated frequent spontaneous termination of ventricular arrhythmias	Immediate therapy overestimates the true need for intervention
**ADVANCE III [10]**	1902 ICD recipients (primary and secondary prevention)	Long detection (30/40) vs. standard (18/24)	Overall ICD therapies reduced by 37% (IRR 0.63; *p* < 0.001); ATP significantly reduced; shocks numerically reduced	No increase in syncope (*p* = 0.22) or mortality (HR 0.87)	Longer observation safely avoids unnecessary ICD therapies
**PainFREE Rx II [11]**	634 ICD recipients with spontaneous VT 188–250 bpm	ATP vs. shock as first therapy	ATP terminated 81% of episodes (72% after adjustment)	No increase in acceleration or syncope	Even fast VTs often remain organized and haemodynamically tolerated

## Data Availability

No new data were generated or analyzed in this study.
